# Lumbar nerve root mapping by the spinal cord monitoring in adult spinal deformity surgery

**DOI:** 10.1186/1748-7161-10-S1-O36

**Published:** 2015-01-19

**Authors:** Tomohiko Hasegawa, Yu Yamato, Daisuke Togawa, Sho Kobayashi, Tatsuya Yasuda, Hideyuki Arima, Yukihiro Matsuyama

**Affiliations:** 1 Dept. of Orthopaedics, Hamamatsu University School of Medicine, Japan

## Objective

When we perform surgical correction for spinal deformity with degenerative changes, nerve root impingement, traction, or nerve root injury could be occurred. Electromyographic (EMG) monitoring provides a real-time measure of impending spinal nerve root injury during instrumented posterior lumbar fusion. However, the utility of intraoperative EMG monitoring is still questionable because of large variance in muscle domination, morphological abnormality and the abnormal EMG signal from a nearby muscle. The aim of this study was identified clinical mapping of lumbar nerve root motor domination under EMG monitoring in the patients who had normal root morphology.

## Materials and methods

We performed corrective surgery for 24 thoracolumbar kyphoscoliosis patients (3 males and 21 females). Average age was 68 y.o. (37-83). All patients underwent the spinal correction by using Ponte osteotomies from L2/3 to L5/S1. They had no severe muscle weakness preoperatively. We exposed bilateral L2-S1 roots and checked root morphology during surgery. Then, we stimulated bilateral L2-S1 roots with 1-10mA by bipolar probe and measured the amplitude of EMG waves in quadriceps femoralis (QF), adductor longus (AL), hamstrings (Ham), tibialis anterior (TA) and gastrocnemius (GC). The first and the second largest EMG amplitudes were investigated in each muscle.

## Results

There was no abnormality of nerve root morphology. We could detect the largest EMG waves in 202 roots (L2:32, L3:45, L4:40, L5:41, S1:44). The second largest wave was detected in 120 roots. The largest EMG wave was observed by L2: QF80%, L3: QF63%, L4: QF45%, L5: TA52% and S1: GC44%. The second largest waves were observed by L2: QF45%, L3: QF48%, L4: QF30%, L5: TA45% and S1: GC41%. The waves of AL were found similar frequency (12-18%) in L2-5, and waves of hamstrings were found similar frequency (10-20%) in L2-S1. The rates of coincidence with right and left roots domination were L2:63%, L3:44%, L4:15%, L5:32%, S1:59%.

## Conclusion

We have investigated the map of lumbar nerve root domination by EMG monitoring. L2, 3 and 4 root stimulation had reacted most frequently in QF. By L5 and S1 stimulations, the EMG waves were detected in TA and GC. AL and Hamstrings were stimulated with similar frequency by several roots. There was a wide variability in muscle domination of lumbar nerve roots. When we use EMG or other neuro-monitoring during lumbar spinal surgery, we have to pay attention to these variability of distributions of muscle domination.

